# Supplementary material for: Genomic structural variations lead to dysregulation of important coding and non‐coding RNA species in dilated cardiomyopathy
Source: EMBO Mol Med. 2017 Nov 14;10(1):107–20. doi: 10.15252/emmm.201707838 (PMC5760848; doi:10.15252/emmm.201707838)
Supplement: Supplementary file 1 — Appendix [file EMMM-10-107-s001.pdf]

Appendix

Genomic Structural Variations Lead to Dysregulation
of Important Coding and Non-Coding RNA Species in Dilated Cardiomyopathy

Jan Haas, PhD[1,4*], Stefan Mester, MSc[1,4*], Alan Lai, PhD[1,4], Karen S. Frese, PhD[1,4], Farbod Sedaghat-Hamedani, MD[1,4], Elham Kayvanpour, MD[1,4], Tobias Rausch, MSc[5], Rouven Nietsch[1], Jes-Niels Boeckel PhD[1,4], Avisha Carstensen MD[1], Mirko Voelkers[1,4], Carsten Dietrich, PhD[2], Dietmar Pils, PhD[6], Ali Amr, MD[1], Daniel B. Holzer[1], Diana Martins Bordalo, MSc[1,4], Daniel Oehler[1,4], Tanja Weis, PhD[1,4], Derliz Mereles, MD[1,4], Sebastian Buss, MD[1], Emil Wirsz, PhD[2], Maximilian Wuerstle, PhD[2], Jan O. Korbel, PhD[5], Andreas Keller, PhD[3], Hugo A. Katus, MD[1,4], Andreas E. Posch, PhD[2], Benjamin Meder, MD[1,4]

[1] Department of Internal Medicine III, University of Heidelberg, Heidelberg, Germany
[2] Siemens Healthcare GmbH, Strategy and Innovation, Erlangen, Germany
[3] Department of Bioinformatics, University of Saarland, Saarbrücken, Germany
[4] DZHK (German Centre for Cardiovascular Research), Germany
[5] EMBL (European Molecular Biology Laboratory), Heidelberg, Germany
[6] Siemens AG, Corporate Technology, Vienna, Austria
* Authors contributed equally to this work

Table of content **Page**

**Appendix Table 1:** Top 20 associated genes from SNV-QTL analysis	3
**Appendix Table 2:** Structural variant eQTL loci	4
**Appendix Table 3:** Differential Expression caused by SVs for functional gene sets	6

Appendix Table 1: Top 20 associated genes from SNV-QTL analysis

Gene Name	ENSEMBL Gene ID	p-value	beta	FDR
HLA-DRA	ENSG00000204287	7.694270×10^{-22}	-4.136061	3.373082×10^{-14}
AGPAT1	ENSG00000204310	2.609624×10^{-19}	-1.908875	4.527451×10^{-12}
HLA-DRB5	ENSG00000198502	1.373991×10^{-18}	-2.906355	4.527451×10^{-12}
HLA-DQB1	ENSG00000179344	3.164864×10^{-18}	-2.615057	4.527451×10^{-12}
HLA-DQA1	ENSG00000196735	3.190135×10^{-18}	-2.288957	4.527451×10^{-12}
RP11-166B2.1	ENSG00000234719	3.799762×10^{-18}	-1.427571	4.527451×10^{-12}
TMEM117	ENSG00000139173	3.829671×10^{-18}	-4.598441	4.527451×10^{-12}
TOM1L1	ENSG00000141198	4.770673×10^{-18}	-4.177049	4.527451×10^{-12}
ERAP2	ENSG00000164308	4.830384×10^{-18}	-2.070406	4.527451×10^{-12}
HLA-F	ENSG00000204642	6.093213×10^{-18}	-2.091995	4.527451×10^{-12}
LDHC	ENSG00000166796	9.327095×10^{-18}	-2.120558	5.181929×10^{-12}
ZC3H10	ENSG00000135482	9.976414×10^{-18}	-3.800280	5.181929×10^{-12}
AASDH	ENSG00000157426	1.251584×10^{-17}	-4.302499	5.751366×10^{-12}
AVIL	ENSG00000135407	3.003591×10^{-17}	-3.268834	1.261246×10^{-11}
PRRC2A	ENSG00000204469	6.334865×10^{-17}	-2.218379	2.552513×10^{-11}
FAM21B	ENSG00000152726	7.860013×10^{-17}	-3.430117	3.155441×10^{-11}
ZNF155	ENSG00000204920	1.320437×10^{-16}	-3.225438	4.586884×10^{-11}
DENND2C	ENSG00000175984	3.613585×10^{-16}	-2.729215	1.124509×10^{-10}
PDIK1L	ENSG00000175087	3.693736×10^{-16}	-4.166681	1.124509×10^{-10}
FABP2	ENSG00000145384	6.789247×10^{-16}	2.4202388	2.030239×10^{-10}

FDR = FDR adjusted p-value

Appendix Table 2: Structural variant eQTL loci

Ensembl ID	GeneName	SV-type	Chr	SV-start	SV-end	min_pval_SNV_eqtl_FDR
ENSG00000070808	*CAMK2A*	INVDEL	5	150319082	150319613	0.0553727407460288
ENSG00000079482	*OPHN1*	DEL	X	66412759	66413050	1
ENSG00000085224	*ATRX*	DEL	X	77121388	77123852	1
ENSG00000086712	*TXLNG*	DEL	X	16805893	16806310	1
ENSG00000099984	*GSTT2*	DEL	22	24274003	24311292	0.00193727130356245
ENSG00000101407	*TTI1*	DEL	20	36311365	36311762	1
ENSG00000105889	*STEAP1B*	DEL	7	21538064	21540560	0.0761151020089635
ENSG00000108349	*CASC3*	DEL	17	38385648	38387943	0.0315762689410888
ENSG00000117226	*GBP3*	DEL	1	89475931	89478608	2.81397292529639e-07
ENSG00000117601	*SERPINC1*	DEL	1	173872947	173886516	0.0856173998178337
ENSG00000121053	*EPX*	DEL	17	57238676	57241299	0.0843994588157762
ENSG00000133433	*GSTT2B*	DEL	22	24274003	24311292	0.00171206712674254
ENSG00000134970	*TMED7*	DEL	5	115531957	115610698	0.00136487415285159
ENSG00000139344	*AMDHD1*	DEL	12	96340185	96342949	0.545898731833455
ENSG00000141433	*ADCYAP1*	DEL	18	828483	828959	1
ENSG00000142528	*ZNF473*	DUP	19	50522237	50592537	4.1891645830034e-07
ENSG00000144671	*SLC22A14*	DUP	3	37751532	37753602	1
ENSG00000145782	*ATG12*	DEL	5	115531957	115610698	0.00111788679229252
ENSG00000155957	*TMBIM4*	DEL	12	66527500	66529870	3.88067681384311e-05
ENSG00000155959	*VBP1*	DEL	X	154485907	154486947	1
ENSG00000155959	*VBP1*	DEL	X	154918462	154921721	1
ENSG00000155962	*CLIC2*	DEL	X	154485907	154486947	1
ENSG00000155962	*CLIC2*	DEL	X	154918462	154921721	1
ENSG00000163596	*ICA1L*	DEL	2	203272279	203279933	0.00525158829466544
ENSG00000167768	*KRT1*	DEL	12	53086343	53087692	0.103227347214968
ENSG00000169914	*OTUD3*	DEL	1	19720443	19732042	0.00141681597663234
ENSG00000169981	*ZNF35*	INVDUP	3	43835086	43836002	1
ENSG00000171084	*FAM86JP*	DEL	3	125672294	125676372	0.0123409136583227
ENSG00000175220	*ARHGAP1*	DUP	11	46742757	46743621	0.37734820366397
ENSG00000178381	*ZFAND2A*	DEL	7	1185026	1187649	0.491554349678595
ENSG00000180438	*TPRXL*	DEL	3	12904009	12914616	0.406584397191934
ENSG00000181541	*MAB21L2*	DUP	4	151292346	151293914	0.601220303762998
ENSG00000184674	*GSTT1*	DEL	22	24274003	24311292	8.32851122310166e-06
ENSG00000185168	*LINC00482*	DEL	17	79143178	79148174	0.166525112807724
ENSG00000188002	*RP11-43F13.1*	DEL	5	1627101	1628833	0.0378748913640489
ENSG00000196646	*ZNF136*	DEL	19	11260612	11272111	0.00164511985536848
ENSG00000198028	*ZNF560*	DEL	19	9274503	9284355	1
ENSG00000204136	*GGTA1P*	DUP	9	124226892	124227825	0.00533577717742678

ENSG00000204209	*DAXX*	DEL	6	33010647	33019605	0.0636799248377916
ENSG00000204619	*PPP1R11*	DEL	6	29685464	29688071	3.0108282921388e-09
ENSG00000204650	*CRHR1-IT1*	DEL	17	44355134	44359562	1.6948442579842e-05
ENSG00000205488	*CALML3-AS1*	DEL	10	5914906	5915914	0.400524998563513
ENSG00000218537	*AP000350.4*	DEL	22	24274003	24311292	0.000556224185176398
ENSG00000221947	*XKR9*	DEL	8	71583568	71584637	0.00653807100311564
ENSG00000224216	*RP13-228J13.1*	DEL	X	154485907	154486947	1
ENSG00000224216	*RP13-228J13.1*	DEL	X	154918462	154921721	1
ENSG00000228701	*TNKS2-AS1*	DEL	10	93633247	93634556	0.000327478049455422
ENSG00000229043	*AC091729.9*	DEL	7	1185026	1187649	0.00047986557356117
ENSG00000230439	*RP11-488P3.1*	DEL	1	94288305	94291249	0.00739322710144155
ENSG00000231482	*AC141930.2*	DEL	2	936820	937135	1
ENSG00000234176	*HSPA8P1*	DEL	X	121138848	121139304	1
ENSG00000235573	*RP3-412A9.12*	DUP	22	32001252	32011923	0.636091195076709
ENSG00000236814	*RP11-446E9.1*	DUP	8	56979007	56979416	0.140735211657874
ENSG00000236829	*Z97634.3*	DEL	16	1290107	1290372	0.0466672753124635
ENSG00000238244	*GABARAPL3*	DEL	15	91219157	91222800	0.00089687726402956
ENSG00000239335	*LLPH-AS1*	DEL	12	66527500	66529870	0.00364890859550425
ENSG00000239445	*ST3GAL6-AS1*	DEL	3	98410646	98414778	0.764433089226613
ENSG00000250007	*RP11-814P5.1*	DEL	15	34717592	34863734	0.103939772677261
ENSG00000253327	*RAD21-AS1*	DEL	8	118910889	118928996	0.0981580849218429
ENSG00000253696	*KBTBD11-OT1*	DEL	8	1305165	1307074	0.696148042545162
ENSG00000253981	*ALG1L13P*	DEL	8	9054963	9062745	0.109212870336974
ENSG00000256278	*RP11-182J1.5*	DEL	15	85475174	85481029	0.0660845877278208
ENSG00000259244	*RP11-182J1.12*	DEL	15	84604908	84606926	0.300033773454947
ENSG00000259592	*PRELID1P4*	DEL	15	34709459	34709717	0.182331373843536
ENSG00000260646	*LA16c-385E7.1*	DUP	16	2186937	2192343	0.151239964949153
ENSG00000260646	*LA16c-385E7.1*	DEL	16	1290107	1290372	0.151239964949153
ENSG00000260923	*AC137934.1*	DEL	16	89967737	89969116	0.143014942049194
ENSG00000261770	*CTC-459F4.1*	DUP	19	27799110	27800050	0.0688959984596499
ENSG00000262902	*RP11-750B16.1*	DEL	17	52158671	52167998	0.104255652967692
ENSG00000264672	*SEPT4-AS1*	DEL	17	57238676	57241299	0.0188921070318906
ENSG00000266235	*MIR3176*	DEL	16	1290107	1290372	0.160588716208391
ENSG00000267179	*ZNF763*	DEL	19	11260612	11272111	0.0397989700457253
ENSG00000269102	*CTD-2525I3.5*	DEL	19	53272779	53277566	0.00778597861927192
ENSG00000272070	*AC005618.6*	DEL	5	140096982	140104134	0.43813848511727
ENSG00000273295	*AP000350.5*	DEL	22	24274003	24311292	4.89377115355646e-09

DEL = large deletion, DUP = large duplication, INVDEL= inversion and a deletion in one locus (complex SV), INVDUP= inversion and a duplication in one locus (complex SV),

Appendix Table 3: Differential Expression caused by SVs for functional gene sets

List	Size	Available	Intersect with SV-eQTL	Determi-nation
ALL HEART EXPRESSED GENES	20712	20712	16449	0.075
GO_MUSCLE_CELL_DEVELOPMENT	88	88	88	0.087
GO_MUSCLE_ORGAN_DEVELOPMENT	182	181	181	0.104
KEGG_ARRHYTHMOGENIC_RIGHT-VENTRICULAR_CARDIOMYOPATHY	54	53	53	0.099
KEGG_CARDIAC_MUSCLE_CONTRACTION	51	51	51	0.098
KEGG_DILATED_CARDIOMYOPATHY	64	64	64	0.101
KEGG_HYPERTROPHIC_CARDIOMYOPATHY_HCM	60	60	60	0.096
