# Supplementary material for: Genomic structural variations lead to dysregulation of important coding and non‐coding RNA species in dilated cardiomyopathy
Source: EMBO Mol Med. 2017 Nov 14;10(1):107–20. doi: 10.15252/emmm.201707838 (PMC5760848; doi:10.15252/emmm.201707838)
Supplement: Supplementary file 2 — Expanded View Figures PDF [file EMMM-10-107-s002.pdf]

Expanded View Figures

Figure EV1. Validation of SV events.

A Plotted are *P*-values for confirmation of SV calls by alternative variant calling from signal intensity values from methylation measurements. The dotted red line indicates a *P*-value ≤ 0.05.

B Shown are PCR-based analyses confirming SVs in the gene loci XKR9, TNKS2-AS1, and KBTBD11-OT1.

Source data are available online for this figure.

© 2017 The Authors

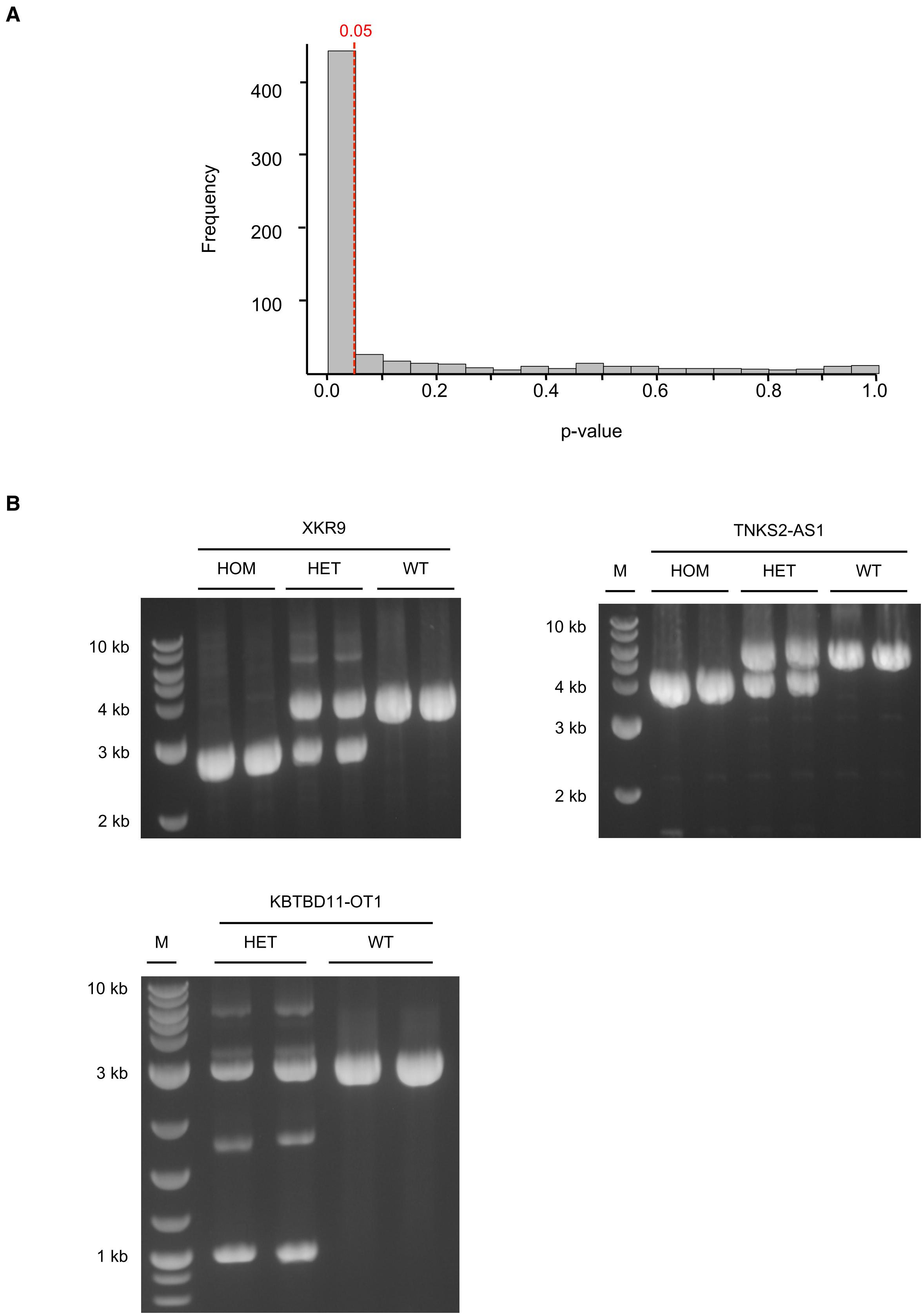

Figure EV1.

© 2017 The Authors

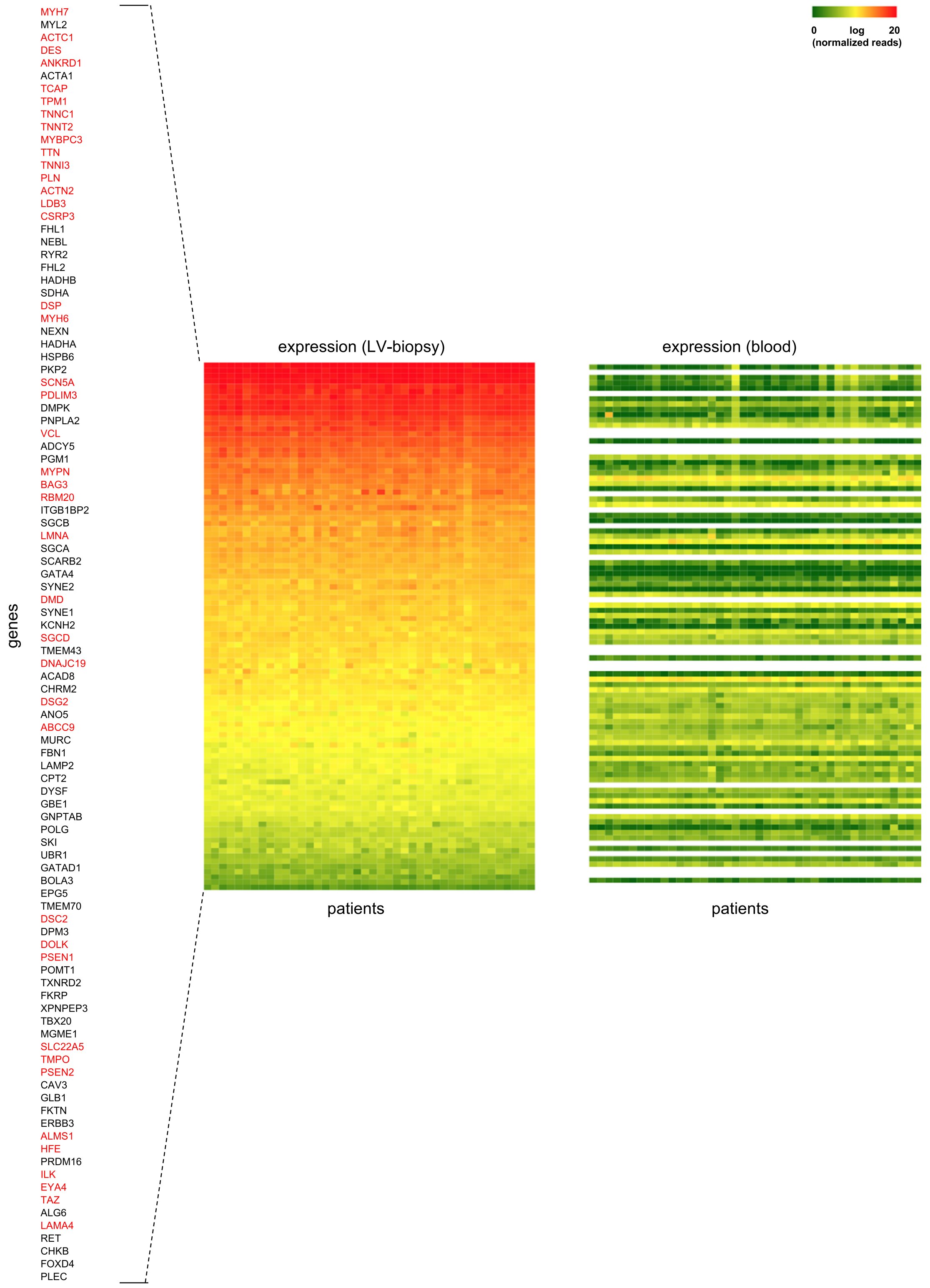

Figure EV2. Gene expression of cardiomyopathy genes.

Shown is the gene expression for genes previously linked to cardiomyopathies in 42 DCM patients as measured by mRNA-seq in left ventricular biopsies (left) and in peripheral blood (right). Major DCM genes are depicted in red and are significantly higher expressed in LV-biopsy and lower expressed in blood compared to other cardiomyopathy genes (black) (Linear regression *P*-value < 0.001). Log (normalized and gene length corrected expression) values range from 0 (green) to 20 (red) and not available (white).

© 2017 The Authors

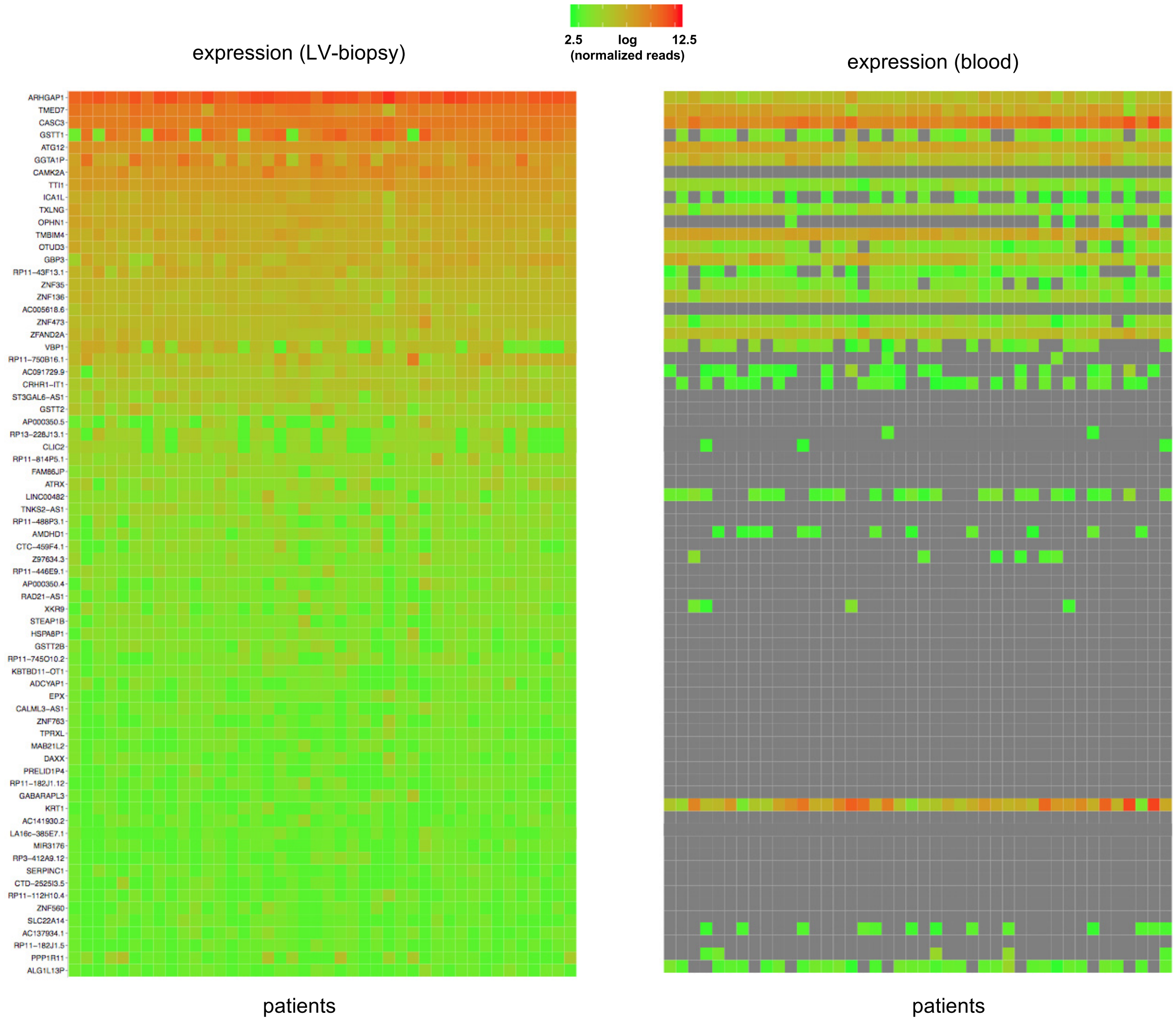

Figure EV3. Gene expression of SV-eQTL genes.

Heatmap showing the cardiac gene expression of 71 genes linked to a structural variant from the eQTL analysis as measured in left ventricular biopsies (left) and in peripheral blood (right). Log(normalized expression) values range from 2.5 (green) to 12.5 (red) and not available (gray).

 © 2017 The Authors

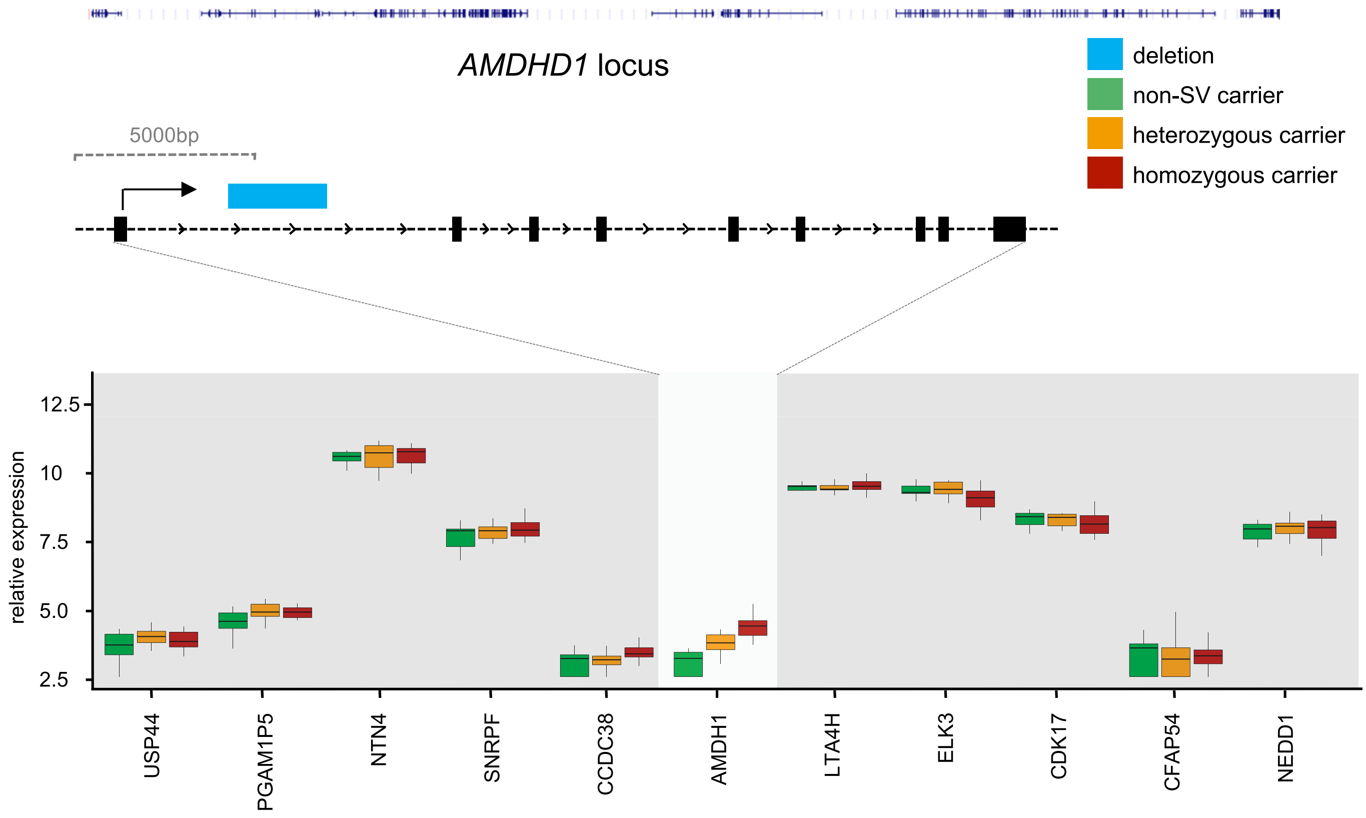

Figure EV4. Genomic context of structural variants of the *AMDHD1* locus.

Shown is a sketch of the genomic structure of genes with aberrant expression due to a linked SV. The deletion (blue) is shown in relation to the exonic structure (black boxes). Below, boxplots are drawn showing the expression of the linked gene and further genes up- and downstream, where patients not carrying the SV (green), patients with a heterozygous SV (orange) or a homozygous SV (red) are separately plotted.

© 2017 The Authors
