# Supplementary figures and images for: Genomic structural variations lead to dysregulation of important coding and non‐coding RNA species in dilated cardiomyopathy
Source: EMBO Mol Med. 2017 Nov 14;10(1):107–20. doi: 10.15252/emmm.201707838 (PMC5760848; doi:10.15252/emmm.201707838)

Figure EV1B

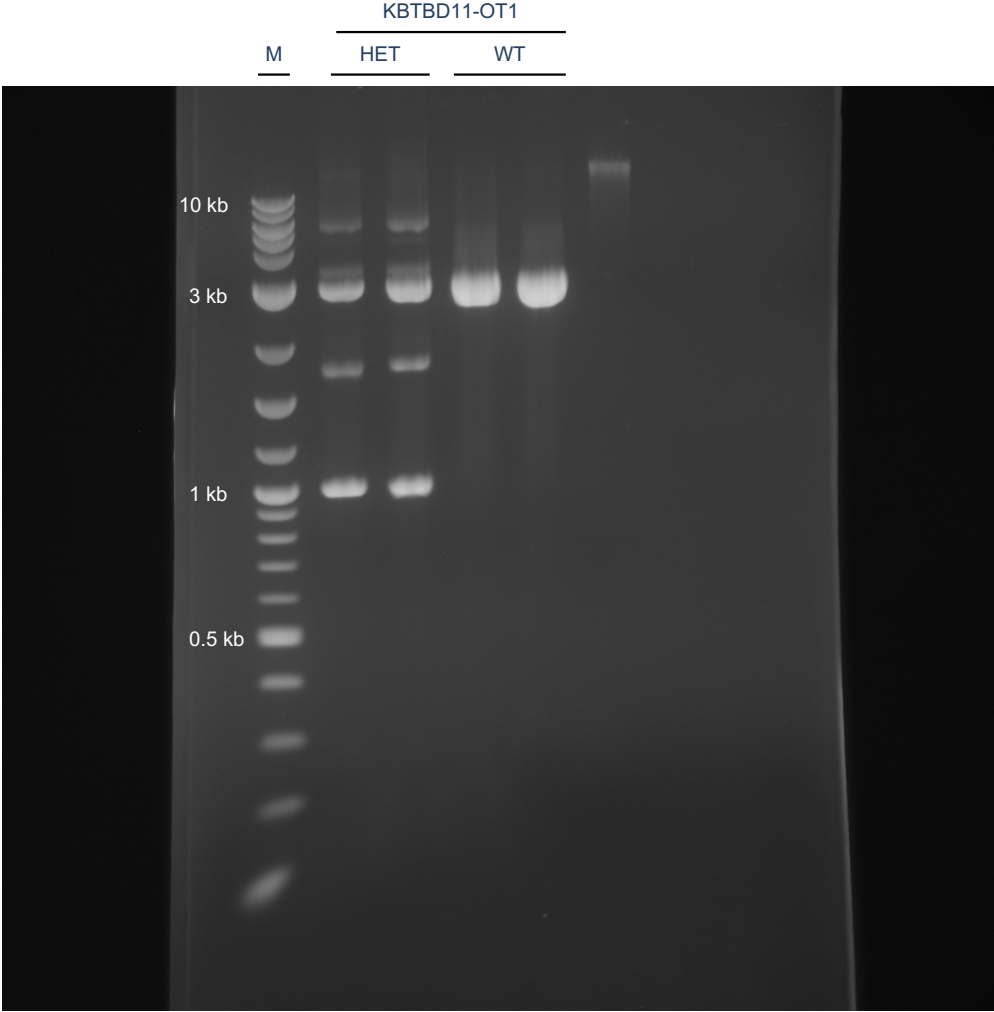

Figure EV1B

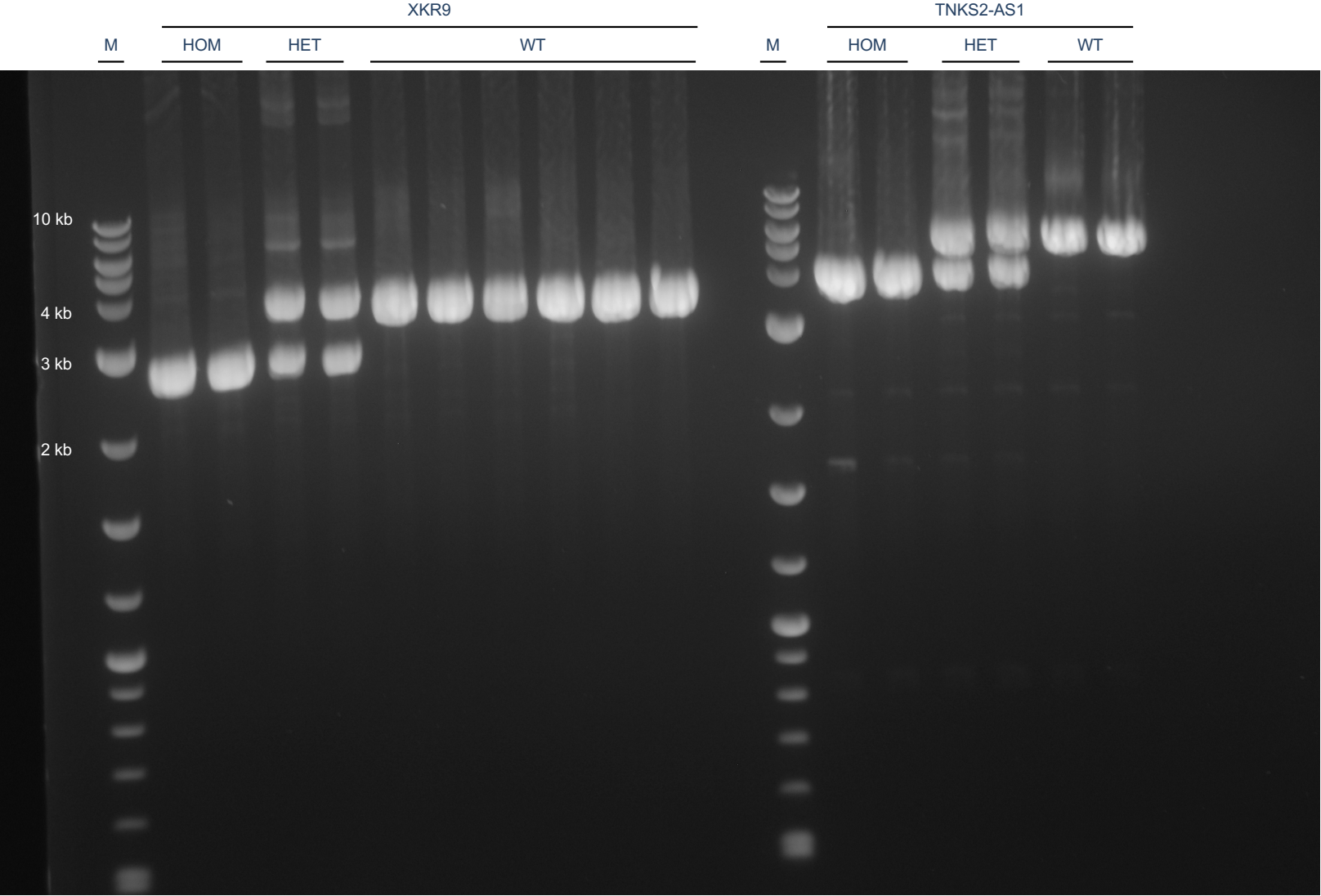

Supplement: Supplementary file 3 — Source Data for Expanded View [file EMMM-10-107-s004.zip › emmm201707838-sup-0004-SDataFigEV1.pdf]

Figure 4B

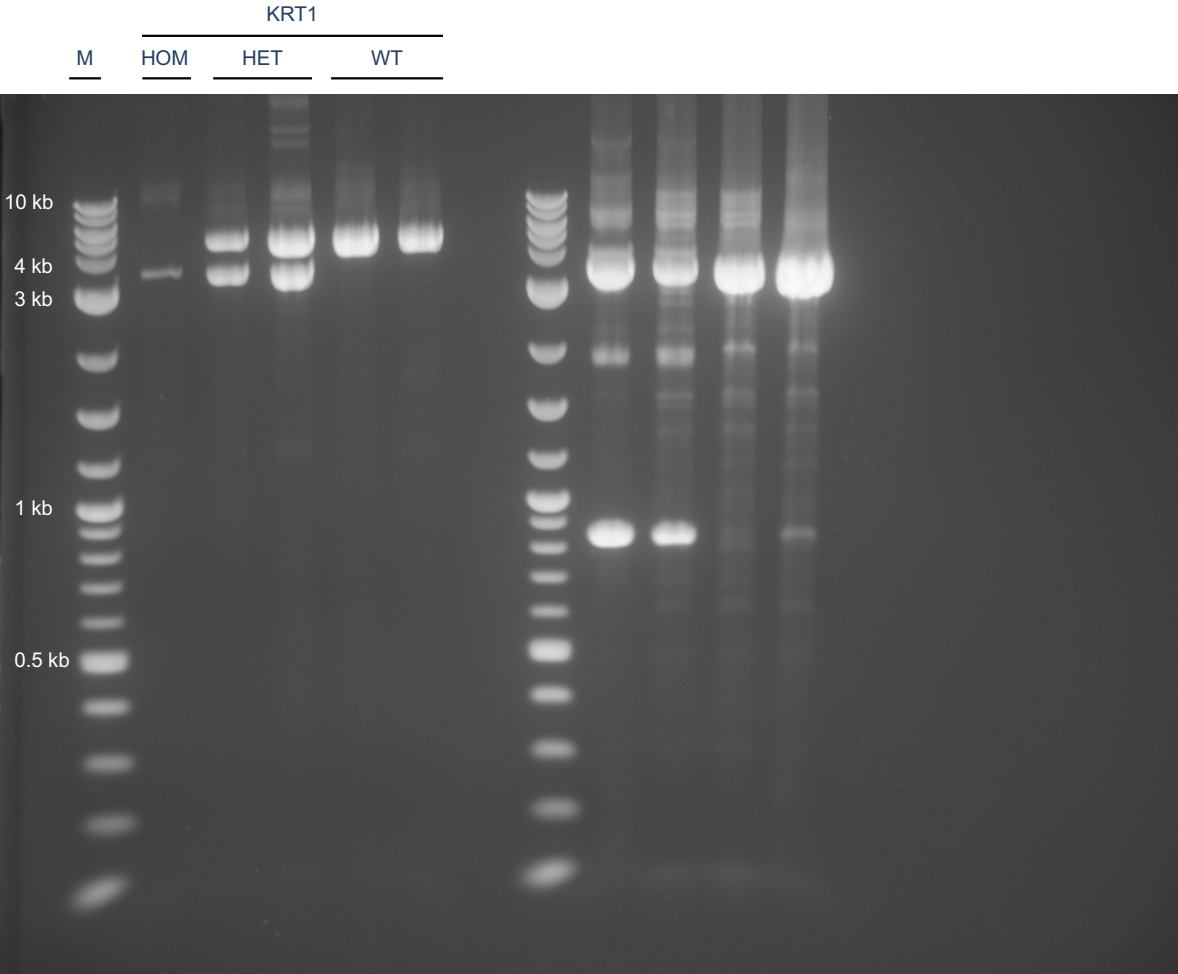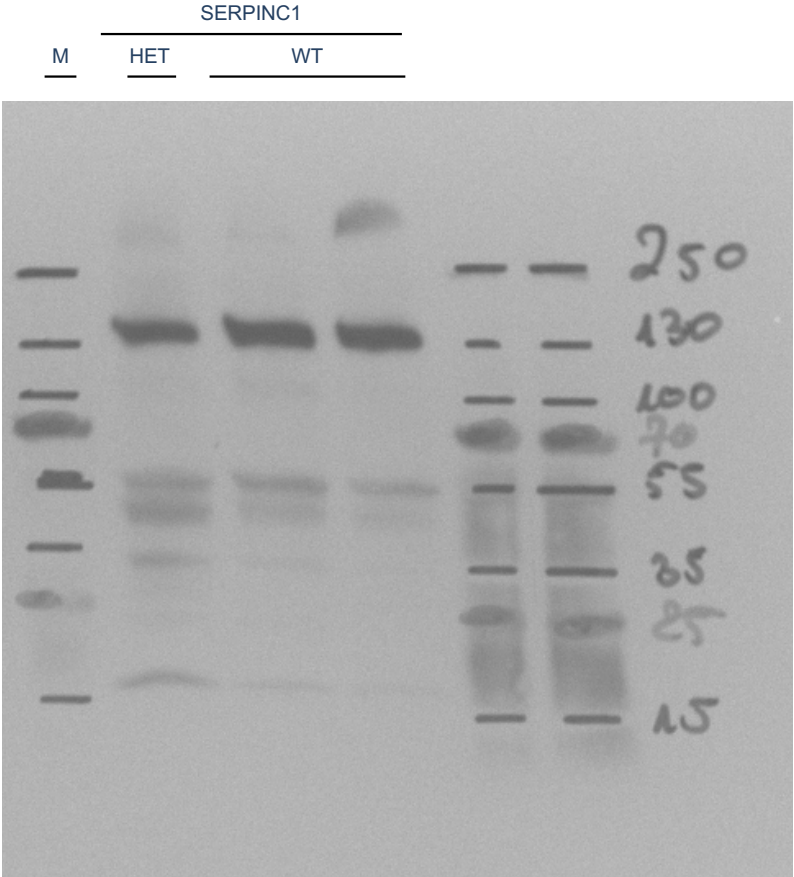

Supplement: Supplementary file 5 — Source Data for Figure 4 [file EMMM-10-107-s003.pdf]
